# Effects of chitosan and TiO_2_ nanoparticles on the antibacterial property and ability to self-healing of cracks and retrieve mechanical characteristics of dental composites

**DOI:** 10.1016/j.heliyon.2024.e27734

**Published:** 2024-03-13

**Authors:** Reza Ravandi, Saeed Zeinali Heris, Salar Hemmati, Marziyeh Aghazadeh, Soodabeh Davaran, Nima Abdyazdani

**Affiliations:** a Faculty of Chemical and Petroleum Engineering, University of Tabriz, Tabriz, Iran; b Drug Applied Research Center, Tabriz University of Medical Sciences, 65811, Tabriz, Iran; c Stem Cell Research Centre and Department of Oral Medicine, Tabriz University of Medical Sciences, Tabriz, Iran; d Department of Medical Nanotechnology, Faculty of Advanced Medical Science, Tabriz University of Medical Sciences, Golgasht St, Tabriz, Iran; e Stem Cell Research Center, Tabriz University of Medical Sciences, Tabriz, Iran

**Keywords:** Dental composites, Chitosan and TiO_2_ nanoparticles, Self-healing, Cytotoxicity

## Abstract

The aim of this study was to improve the self-healing properties of dental nanocomposite using nanoparticles of TiO_2_ and chitosan. We evaluated flexural and compressive strength, crack-healing, and self-healing lifespan after 3 months of water aging. The effect of the developed composite on cell viability and toxicity was assessed by an MTT assay on human alveolar basal epithelial cells (A549 cell line). The nanocomposite included 7.5 wt% polyurea-formaldehyde (PUF) and 0, 0.5, and 1 wt% n-TiO_2_ and chitosan. After the fracture, the samples were put in a mold for 1–90 days to enable healing. Then, the fracture toughness of the healed nanocomposites and the healing yield were measured. The flexural strength of the nanocomposite improved by adding 0.5 wt% n-TiO_2_, while the compressive strength increased after adding 0.5 wt% chitosan (p > 0.1). When these two materials were used simultaneously, the flexural strength was improved by around 2%; however, the compressive strength was unaffected. Compared to the other sample, the nanocomposite with 0.5 wt% n-TiO_2_ and chitosan had higher K_IC-healing_ and self-healing efficiency. Self-healing efficacy had no significant effect of water aging over 90 days compared to one day (p > 0.1), demonstrating that the PUF nanocapsules were not damaged.

## Highlights

• Nanocomposite consists of chitosan & TiO_2_ nanoparticles prepared and characterized.
• Cracks healed autonomously with self-healing dental composites.
• After 90 days immersed in distilled water, self-healing efficiency did not decrease.
• Nanocomposite 0.5 wt% TiO_2_ & Chitosan demonstrated high self-healing retention.
• Regarding cell viability, 0.5 wt% TiO_2_&Chitosan demonstrate perfect biocompatibility.

## 1 Introduction

Tooth decay is one of the most common chronic disorders in humans globally [1,2]. Resin composites (RCs) have been widely used in dental restoration due to their appealing clinical performance, such as aesthetics, lifespan, low cost, non-toxicity, and simplicity of clinical manipulation [3,4]. The development of dental composites using nanomaterials has caused rapid growth in dental research because they can improve biological and mechanical properties [[5], [6], [7], [8]]. However, restoration fractures and secondary caries affect its clinical applicability [[9], [10], [11]]. The major cause of secondary caries is bacterial development on the surface of the resin composite [12,13], which can be efficiently reduced by improving the antibacterial capabilities of the dental composite [[14], [15], [16]]. Antibacterial materials are classified into two types: inorganic and organic materials. Inorganics include metal oxides, metals, and metal phosphates [17]. Various kinds of metal-oxide nanoparticles, such as titania (TiO_2_), CaO, ZnO, ZrO_2_ and metallic nanoparticles such as silver (Ag), are linked for use in the fabrication of dental nanocomposite [[18], [19], [20], [21], [22]]. Chitosan (CH) is one of the most widely used organic antibacterial materials in dentistry [23,24]. Chitosan is a natural linear polysaccharide produced by deacetylating chitin derivatives [25]. Chitosan's characteristic properties are biodegradability, nontoxicity, cost-effectiveness, bio-adhesiveness, biocompatibility, and bio-renewability, which have led to its widespread use in dentistry for osteogenesis, edentulous ridge augmentation, as a guided bone regeneration tool, and salivary secretion stimulation [23]. Furthermore, chitosan has a high resistance to heat due to its intramolecular hydrogen bonding [26,27].

Incorporating TiO_2_ nanoparticles in the structure of dental composites caused an improvement in strength, effect resistance due to body fluid, and corrosion resistance while also providing antibacterial properties [[28], [29], [30]]. These particles, when incorporated into polymers like dental adhesives, can function as co-initiators in the photo-polymerization reaction [31,32]. Furthermore, TiO_2_ demonstrates bioactivity by forming a surface of calcium phosphates, which enhances the stability of dental adhesives in long-term restorative treatments [33,34].

RCs utilized as fillings in dentistry are vulnerable to damage such as frequent dynamic mechanical stress, microcracks produced by polymerization shrinkage, water sorption, and thermal fatigue. These tensions might be the main cause of the failure. As a result, preventing crack propagation and fracture in resin-based restorations is seen as crucial [[35], [36], [37]]. The development of self-healing dental resins that repair cracks as long as they restore stress is an innovative method [38]. In this method, microcapsules are incorporated into a resin matrix, each of which has an external shell containing a healing liquid [39]. After microcracks emerge in the resin matrix, the microcapsules rupture and release the healing liquid [40]. Then, the healing liquid moves into the fracture planes, exposing the catalyst in the resin, which ends in crack polymerization [41]. To produce microcapsules, dicyclopentadiene (DCPD) was enclosed in a polyurea-formaldehyde (PUF) shell [[42], [43], [44]]. The average rate of recovery of the fracture toughness was 57% after the incorporation of a self-healing microcapsule [28,36]. Adding microcapsules had no effect on the mechanical properties of dental resins [39]. The toxicity and high expense of Grubb's catalyst, as well as the toxicity of DCPD, prevented their usage in dental composites [45]. In recent research, crack-healing PUF microcapsules, N,N-Dimethyl-*p*-toluidine (DEPT), and TEGDMA were synthesized [31]. Previous research on dental nanocomposites that do not self-heal has demonstrated that long-term water aging weakens the fillers and causes the degradation of the composites [38]. In this research, we incorporated n-TiO_2_ and chitosan into the self-healing dental nanocomposites and investigated their effects on compressive and flexural strength, water-aging, and biomedical properties.

## 2 Material and method

### 2.1 Materials

Nano-titanium oxide, ammonium chloride (NH₄Cl, 99%), resorcinol (C₆H₆O₂, 99%), sodium hydroxide (NaOH, 98%), urea (CH₄N₂O, 99%), poly(ethylene-*alt*-maleic anhydride) (EMA), N,N-dihydroxyethyl p-toluidine (DEPT, 90%)), and benzoyl peroxide (BPO, 98%) were obtained from Merck. Formaldehyde solution (37%), bisphenol A-glycidyl methacrylate (Bis-GMA, 97%), triethylene glycol dimethacrylate (TEGDMA, 95%), camphorquinone (C_10_H_14_O_2_, 97%), urethane dimethacrylate (UDMA, 97%), nanoclay, and nano-chitosan were purchased from Sigma-Aldrich.

### 2.2 PUF nanocapsules preparation

In situ polymerization of urea via formaldehyde was used to produce PUF nanocapsules. A mixture of 99 wt% TEGDMA and 1 wt% DEPT monomer was prepared and added to a 200-mL round-bottom Erlenmeyer flask containing 50 mL of distilled water and 13 mL of a 2.5 percent aqueous EMA copolymer at ambient temperature. In order to create an "oil-in-water" emulsion, the EMA solution was utilized as a surfactant. To form the shells, 0.125 g of resorcinol, 0.125 g of ammonium chloride, and 1.25 g of urea were added to the solution while it was stirring at 300 rpm. Resorcinol was incorporated during the shell production process to enhance the stiffness of the shells. Add 1 M of sodium hydroxide solution dropwise to adjust the pH value to 3.5. In the flask, 30 mL of DEPT-TEGDMA liquid was combined while the agitation speed was increased to 1300 rpm. 15 min of stirring resulted in the formation of a stable emulsion of fine DEPT-TEGDMA droplets. To avoid evaporation, the flask was covered with aluminum foil after adding 3.2 g of a 37% aqueous formaldehyde solution. The substance in the shell was isothermally polymerized in a 55 °C water bath for 4 h with continual agitation. Ammonium chloride catalyzed the reaction of urea and formaldehyde to produce PUF at the oil-water interface. The synthesized PUF nanocapsules were washed with distilled water and acetone, filtered with a vacuum, and air-dried for one day [45].

### 2.3 Preparation of self-healing dental nanocomposites with n-TiO_2_ and chitosan

The methacrylate monomers were produced by mixing Bis-GMA/UDMA/TEGDMA in the following ratios: 7:2:1 [[45], [46], [47]]. Three concentrations of n-TiO_2_ (0, 0.5, and 1 wt%) and three concentrations of chitosan (0, 0.5, and 1 wt%) were mixed with 7.5 wt% of synthesized PUF nanocapsules and 53 wt% nanoclay (with the addition of n-TiO_2_ and chitosan, the weight percentage of nanoclay was reduced). To achieve a homogenous resin blend, the fillers were blended with an acrylate monomer combination. Half of this compound was combined on the various glass surfaces with 0.1 wt% DEPT, while the other half was combined with 0.4 wt% BPO and 0.1 wt% camphorquinone photoinitiator. In this method, BPO was used for chemical curing and camphorquinone for light curing, causing a dual cure. As an initiator, Self-healing polymerization is initiated by the reaction between the BPO in the mixture and DEPT encapsulated within the PUF nanocapsules. Before being inserted into molds, these two combinations were completely and rapidly (3 min) mixed on the work glass surface.

### 2.4 Characterization

SEM was utilized to characterize the morphology of samples coated with gold (SEM, Model MIRA3 Tescan). The samples' compressive strength was determined using a universal testing apparatus (Hounsfield Test Equipment) at a cross-head speed of 0.5 mm/min. Utilizing universal testing apparatus, the samples' three-point flexural test was conducted (Hounsfield Test Equipment).

### 2.5 Measurement of compressive and flexural strength

The ISO 4049-compliant compressive strength test was performed. Eight samples (from each nanocomposite) were manufactured by filling split-ring Teflon molds (3 mm × 6 mm) with excess mixed composites, compressing them between two glass slabs, and curing them for 10 min. Samples were analyzed at a cross-head speed of 0.5 mm/min after storage for one day at 50 °C in an oven and a 50 kN load using Instron test equipment (Hounsfield Test Equipment, Model H5KS-Surrey-UK). Equation (1) was utilized to determine the compressive strength (MPa) [45]:

(1) $$\text{Compressive}\;\text{strength}=\frac{F}{A}$$

F (N) denotes the failure load, and A (mm^2^) is the cross-sectional area.

The flexural strength of eight samples (from each nanocomposite) was measured and composited into a rectangular stainless-steel mold (2 × 2 × 25 mm^3^) according to ISO 4049. Equation (2) was used to calculate flexural strength (MPa), where F denotes the failure load (N), L denotes the distance between supports (mm), b denotes the sample width (mm), and h denotes the sample thickness (mm) [44,46].

(2) $$\text{Flexural}\;\text{strength}=\frac{3\text{FL}}{\left(2bh^{2}\right)}$$

### 2.6 Measurement of self-healing efficiency and K_IC_ test

The fracture toughness (K_IC_) was calculated utilizing the SEVNB (single edge V-notched beam) method. A thin diamond blade with a thickness of 150 μm (Buehler) was used to machine a 500 μm thickness notch in a rectangular cube measuring 2 × 2 × 25 mm. Following this, the notch tip was filled with diamond paste with a particle size of 3 μm, and a new razor blade was employed to cut the notch to a total depth of approximately 700–800 μm. This method produced a sharp notch tip of roughly 20 μm. After notching them with a razor blade and suspending them in 3 μm diamond suspensions, the fracture toughness of rectangular cubes was assessed using the three-point flexure test. This procedure produced a 20 μm sharp notch tip. The flexure rectangular cubes were notched using a razor blade, and subsequently, they were suspended in 3 μm diamond suspensions. The fracture toughness was evaluated using the same three-point flexure test. This produced the sample's virgin fracture toughness, indicated as K_IC-virgin_. Hence, the virgin fracture toughness of the material, denoted as K_IC-virgin_ was obtained. To evaluate self-healing efficiency, two halves of the nanocomposite were placed rapidly back into the mold after the sample fractured to enable a good connection between the two fractured planes. By releasing healing fluids, the fractured nanocomposite at a tooth-restoration contact healed the crack. The DEPT-TEGDMA released from the ruptured PUF nanocapsules reacts with the BPO in the combination. The released liquid would polymerize, binding and healing the two fracture planes together to form a single cohesive sample. The healing specimens were put in a humidor at 37 °C for one day. The K_IC-healed_ was evaluated, and the healing yield was determined. Equation (3) was utilized to calculate the efficacy of self-healing (η) [45,48].

(3) $$\eta =\frac{K_{IC-healed}}{K_{IC-virgin}}$$

### 2.7 Measurement of water-aging on nanocomposites

The flexural strength of the self-healing sample was increased by adding n-TiO_2_ and chitosan from 0 to 0.5 wt% compared to the virgin sample, while raising the amount of them to 1 wt% caused reduced flexural strength. Consequently, the nanocomposite containing 0.5 wt% n-TiO_2_ and chitosan was selected for a 90-day water aging test. Cured samples with diameters of 2 × 2 × 25 mm were immersed in distilled water for 1 day, 15, 30, and 90 days at 37 °C. Eight samples were immersed in 200 mL of water in a sealed container for each period. Once a week, the water was changed. K_IC-healed_ is evaluated after each aging period [45].

### 2.8 MTT assay

The cell compatibility of the nanoparticles was evaluated by the 3-(4,5-Dimethylthiazol-2-yl)-2,5-diphenyltetrazolium-bromide (MTT) test on the A59 cell line. The cells were expanded, cultured in a 96-well plate, and treated with various concentrations of n-TiO_2_ and chitosan. 5% Triton X-100 and culture media were used as the positive and negative controls, respectively. MTT solution was added to each well 24 and 48 h after treatment, and the plates were then incubated for 3 h at 37 °C. MTT, which is soluble in water, is transformed into insoluble formazan by succinate dehydrogenase within the mitochondria via tetrazolium ring cleavage. The formazan product is cell membrane impermeable and accumulates in healthy cells. Because formazan is purple, the change in color of the solution indicates cellular activity. After discarding the supernatants, 100 μL of DMSO was added to each well and incubated for 20 min. The optical density (OD) of each well was determined at a wavelength of 570 nm. Equations (4), (5)) were utilized to calculate the percentages of cytotoxicity and cell viability, respectively [46].

(4) Cellcytotoxicity(%)=(1−cellviability)×100

(5) $$\text{Cell}\;\text{viability}\;\left(\%\right)=\frac{\text{Mean}\;\text{absorbance}\;\text{of}\;\text{toxicant}-\text{OD}\;\text{blank}}{\text{Mean}\;\text{absorbance}\;\text{of}\;\text{negative}\;\text{control}\;-\;\text{OD}\;\text{blank}}\times 100$$

### 2.9 Statistical analysis

Analysis of Variance (ANOVA) is a statistical tool used to determine each factor's importance in an outcome. Tukey's multiple comparisons were used to evaluate and compare compressive and flexural strength values using a one-way ANOVA test at a p value of 0.05.

## 3 Result

### 3.1 Morphological properties of self-healing dental nanocomposites

**Fig. 1:**
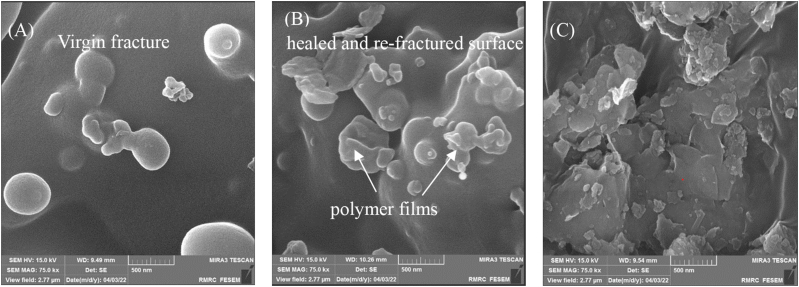
SEM image of the fractured surfaces of nanocomposite comprising 0.5 wt% n-TiO_2_ and chitosan (A) surface of virgin fracture and (B) surface of fractured, healed, and re-fractured. (C) surfaces of nanoclay.

Fig. 1 (A) demonstrates a SEM micrograph of a delegate nanocomposite with fracture surfaces including 7.5 wt% PUF nanocapsule, 0.5 wt% n-TiO_2_, and 0.5 wt% chitosan. This image demonstrated the presence of large porous structures, which might be attributed to particle agglomeration and non-homogeneous particle distribution in the nanoclay matrix, leading to weak spots in the structure. The PUF nanocapsules released TEGDMA-DHEPT liquid, which reacted with the BPO and caused in situ polymerization in the resin matrix. SEM micrographs of the fractured, healed, and re-fractured surfaces are shown in Fig. 1(B). Various irregular-shaped polymer films (arrows) are observed on the fracture surface in Fig. 1 (B), demonstrating that the DEPT-TEGDMAwas released and polymerized. Fig. 1 (C) shows a SEM image of a nanocaly. Nanoclays have a high porosity, a large surface area, and a small particle size.

### 3.2 Effect of n-TiO_2_ and chitosan on the mechanical properties of nanocomposites

**Fig. 2:**
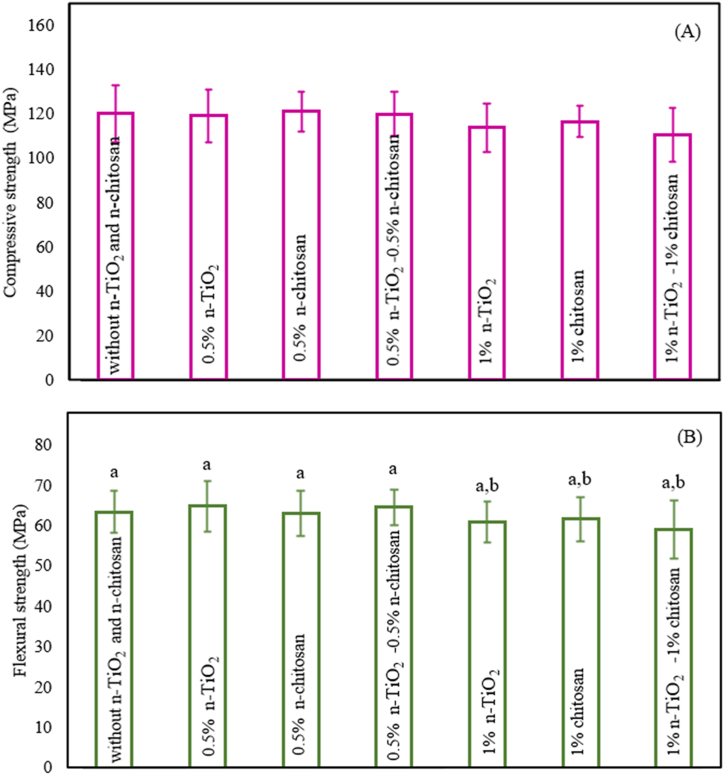
(A) Compressive strength of dental nanocomposites, containing 0, 0.5, and 1% n-TiO_2_ and chitosan, (B) Flexural strength of dental nanocomposite, containing 0, 0.5, and 1% n-TiO_2_ and chitosan, after 1 day of water immersion.

One of the goals of adding n-TiO_2_ and chitosan to the nanocomposite is to investigate their effect on the mechanical strength of the nanocomposite. The compressive strength of dental nanocomposites containing 7.5 wt% PUF with 0, 0.5, and 1 wt% n-TiO_2_ and chitosan is depicted in Fig. 2 (A) (n = 8; mean ± sd), which is the standard deviation from the eight sample repetitions. Table 1 indicates that the incorporation of 0.5 wt% of both n-TiO_2_ and chitosan has a different effect on the mechanical characteristics of the nanocomposite. Specifically, the addition of 0.5 wt% of n-TiO_2_ results in a slight decrease in compressive strength up to 0.7%, while the incorporation of the same percentage of chitosan leads to an increase of almost 0.6%. However, when both materials are used together, their effects counterbalance each other, and the compressive strength of the nanocomposite remains unchanged. On the other hand, the addition of 0.5 wt% of n-TiO_2_ amplifies the flexural strength of the nanocomposite by 2.3%, while the addition of 0.5 wt% of chitosan reduces it by 0.5%. Nevertheless, the simultaneous usage of both materials at 0.5 wt% leads to an increase in the flexural strength of the nanocomposite of 1.9%. Increasing the weight percentage of both n-TiO_2_ and chitosan to 1 results in a reduction of the compressive and flexural strengths of the nanocomposite. Therefore, the flexural and compressive strengths decrease with the addition of n-TiO_2_ and chitosan at a higher percentage. Therefore, it can be concluded that the optimum weight percentage of n-TiO_2_ and chitosan in the nanocomposite is 0.5 for improving its mechanical properties. n-TiO_2_ can form strong bonds with the matrix material of the composite, enhancing the interfacial adhesion. This improved interaction between the filler and the matrix can enhance the flexural strength. On the other hand, if n-TiO_2_ agglomerates or clusters together within the composite material, it can create regions of weakness and reduce the surface hardness and compressive strength. Agglomeration can lead to stress concentration points, making the material more susceptible to deformation or failure under compressive loads. Values with distinct letters exhibit significant differences from each other (p < 0.05), as seen in Fig. 2 (B) for the letter "b". On the other hand, values with similar letters are nearly equivalent and show no significant difference (p > 0.1), as demonstrated by the letter "a" in Fig. 2 (A).

**Table 1:** Effect of n-TiO_2_ and chitosan on Compressive strength and flexural strength of dental nanocomposite.

Number	Sample	Compressive strength (MPa)	Flexural strength (MPa)
1	0% TiO_2_, 0% Chitosan	120.12 ± 1.3	63.31 ± 1.0
2	0.5% TiO_2_, 0% Chitosan	119.20 ± 1.2	64.75 ± 1.2
3	0% TiO_2_, 0.5% Chitosan	121.02 ± 0.9	62.98 ± 1.1
4	0.5% TiO_2_, 0.5% Chitosan	119.94 ± 1.0	64.50 ± 0.9
5	1% TiO_2_, 0% Chitosan	113.73 ± 1.1	60.88 ± 1.0
6	0% TiO_2_, 1% Chitosan	116.62 ± 0.7	61.57 ± 1.1
7	1% TiO_2_, 1% Chitosan	110.61 ± 1.2	59.06 ± 1.4

### 3.3 Effect of n-TiO_2_ and chitosan on self-healing efficiency and K_IC_ test

**Fig. 3:**
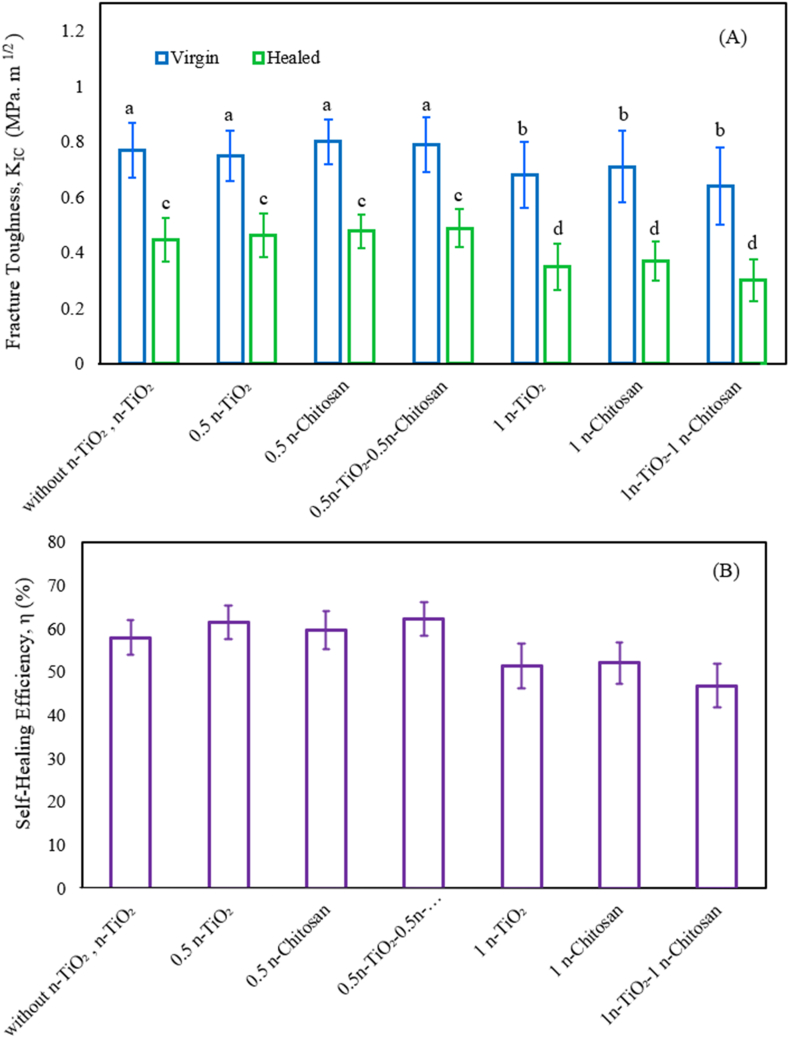
Self-healing nanocomposite containing 0, 0.5, and 1% n-TiO_2_ and chitosan for 1 day: (A) Fracture toughness; (B) Efficiency of self-healing.

The K_IC-virgin_ and K_IC-_ _healed_ resins after adding a variable amount of n-TiO_2_ and chitosan for 1 day are shown in Fig. 3 (A). Incorporating 0.5 wt% n-TiO_2_ to the resin decreased the K_IC-virgin_ from 0.77 MPa m^1/2^ to 0.75 MPa m^1/2^ compared to virgin nanocomposite (p < 0.05), while adding 0.5 wt% chitosan to the resin caused an increase in the virgin K_IC_ to 0.80 MPa m^1/2^. However, when both materials were used together, the virgin K_IC_ increased to 0.79 MPa m^1/2^. Increasing the weight percentage of both n-TiO_2_ and chitosan to 1 resulted in a reduction in the virgin K_IC_ of the nanocomposite. Therefore, the virgin K_IC_ decreased due to the addition of n-TiO_2_ and chitosan at this higher percentage. Furthermore, the K_IC-healed_ valve increased from 0.44 MPa m^1/2^ to 0.46 MPa m^1/2^ with the addition of 0.5 wt% n-TiO_2_, and the addition of 0.5 wt% chitosan increased the K_IC-healed_ to 0.45 MPa m^1/2^. When both materials are used together, the K_IC-healed_ increased to 0.488 MPa m^1/2^. However, increasing the weight percentage of n-TiO_2_ and chitosan to 1% caused a large decrease in the K_IC-healed_ and significantly reduced the self-healing efficiency, as demonstrated in Fig. 3(B).

### 3.4 Effect of n-TiO_2_ and chitosan on water-aging

**Fig. 4:**
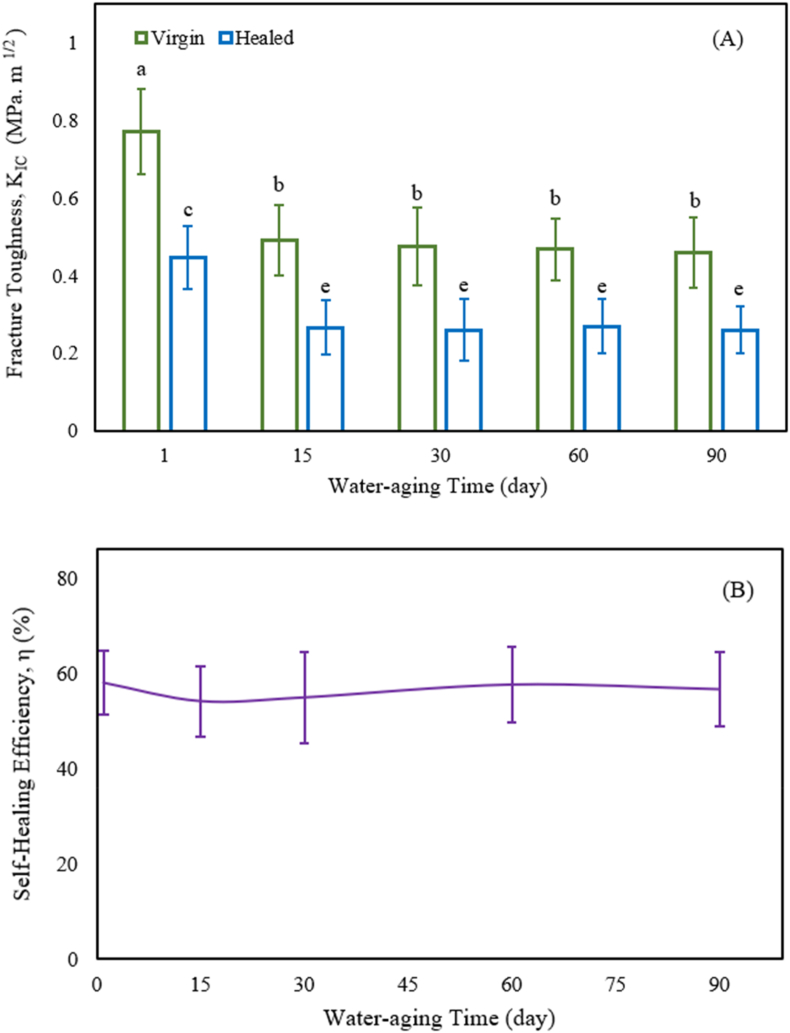
Effect of 0.5 wt% n-TiO_2_ and chitosan in self-healing dental nanocomposite (A) Fracture toughness and (B) efficiency of self-healing on water aging from 1 to 90 days.

The samples of cured nanocomposites adding 0.5 wt% n-TiO_2_ and chitosan were water-aged for 3 months and then evaluated for self-healing. Fig. 4 demonstrates the effect of water aging on (A) fracture toughness before and after healing and (B) self-healing efficiency (n = 8; mean ± sd). K_IC-virgin_ for nanocomposite containing 0.5 wt% n-TiO_2_ and chitosan compared to the sample without n-TiO_2_ and chitosan increased by about 6.5% in 1 day, while water-aging for 1–15 days significantly decreased the K_IC-virgin_. After 15 days of water ageing, the K_IC-virgin_ of the nanocomposite was 0.574 MPa m^1/2^, a 30% decrease from its initial value. K_IC-virgin_ decreased a little as the immersion time was extended from 15 to 90 days, and K_IC-virgin_ was slightly reduced. The pattern observed in K_IC-healed_ closely resembled the pattern observed in K_IC-virgin_. In Fig. 4 (B), the healing efficiency varied from 58.46 to 62.31%, with no significant decreases from 1 to 90 days (p > 0.1). After 90 days of water aging, the self-healing efficiency of 59.84% indicated that the self-healing durability was good. The dental composite used in this study has a pH of roughly 7, which is in the range of 6.2–7.6 for the mouth. When compared to buffers and tampons, there is no need to investigate the effect of additional ions in distilled water (pH = 7).

### 3.5 Effect of n-TiO_2_ and chitosan on A549 cell line viability

The decrease in antibacterial properties of nanocomposite with increasing TiO_2_ concentration can be due to one of the following reasons: Adding more TiO_2_ to dental composites could weaken the bond between the matrix and filler and reduce the antibacterial properties of the nanocomposite. Some low-quality TiO_2_ types may make the composite more susceptible to corrosion and discoloration and reduce its antibacterial property. Incorporating TiO_2_ as a filler in the presence of oxygen and light may result in free radicals that react with the carbon matrix or filler, reducing the composite's antibacterial properties.

The MTT assay was utilized to investigate the effects of various concentrations of n-TiO_2_ and chitosan in the nanocomposite on in vitro cell viability after 24 and 48 h. As shown in Table 2, adding 0.5 wt% of chitosan and n-TiO_2_ had the highest performance in enhancing cell proliferation compared to other concentrations. Furthermore, increasing concentrations of n-TiO_2_ and chitosan led to a decrease in cell proliferation.

**Table 2:** Cell viability percentage at different concentrations of chitosan-TiO_2_ nanocomposite.

	Viability % (24 h)	p-value	Viability % (48 h)	p-value
control	100		100	
Without PUF	100	P < 0.001	100	P < 0.001
With PUF	95.8	P < 0.05	95.1	P < 0.05
0.5% chitosan	99.2	P < 0.001	98.7	P < 0.001
0.5% TiO_2_	97.8	P < 0.01	97.1	P < 0.01
0.5% chitosan-0.5% TiO_2_	100	P < 0.001	100	P < 0.001
1% chitosan	97.2	P < 0.01	93.6	P < 0.01
1% TiO_2_	91.2	P < 0.01	84.3	P < 0.05
1% chitosan-1 TiO_2_	87.5	P < 0.05	73.7	P < 0.05

This research investigated the effect of adding n-TiO_2_ and chitosan on the mechanical characteristics, crack healing, and antibacterial properties. Nanoclay was widely used as an inorganic filler in polymer applications for its ability to substantially increase and modify the mechanical characteristics of dental nanocomposites, such as an increase in flexural strength and a reduction in crack propagation.

Recently, the effect of various particle size distributions of PUF nanocapsules and water-aging on the self-healing properties of dental nanocomposites was investigated [49]. A nanocomposite comprising 7.5% PUF nanocapsules was plunged in 37 °C distilled water for 1–90 days to assess aging. The DHEPT-TEGDMA healing agent is released, and polymerization initiates when the encapsulated microcapsules fracture. The results demonstrated that after three months, the K_IC-virgin_ did not significantly decrease [45]. However, no research has been performed on the effect of chitosan/n-TiO_2_ and nanoclay as fillers in self-healing dental composites.

Two factors related to the self-healing mechanism may have led to these findings. Firstly, the cured TEGDMA-BisGMA composite matrix has a hydrophobic surface. Water infiltration into the crack is thus made more difficult. Secondly, water cannot prevent the self-healing polymerization process. Because water cannot enter the chemical structure of the resin, the catalyst-initiator reaction takes place within. The polymerization would not be impacted by the presence of water. The degree of double bond conversion, or rate of photo-polymerization, is little affected by water [49].

The rupture technique of the encapsulated PUF nanocapsules resulted in high self-healing efficiency, polymerization in the resin matrix with reacting BPO, and the release of the released DEPT-TEGDMAhealing liquid. The SEM images show that ruptured PUF nanocapsules and the released TEGDMA-DEPT, which reacted with the BPO, resulted in autonomous healing and fracture repair in nanocomposites. The flexural and compressive strengths for the nanocomposite comprising 0.5 wt% of chitosan and n-TiO_2_ are about 64.50 MPa and 119.94 MPa, respectively, which shows that it has no impact on the compressive strength (p > 0.1) but increases the flexural strength of the nanocomposite by around 2% (p < 0.05). In this study, self-healing efficiency was obtained at roughly 62.31% after 90 days of water aging, indicating that the self-healing procedure was not significantly affected by water aging (p > 0.1). As a result, it is expected that this biocompatible and self-healing composite will be useful in dental restorative fractures.

## 4 Conclusions

In this research, a self-healing dental nanocomposite comprised of chitosan and n-TiO_2_ has been evaluated for compressive and flexural strength, long-term water aging, crack self-healing ability, and MTT tests for toxicity. The SEM image demonstrated autonomous nanocomposite repair and crack healing after the PUF nanocapsules were ruptured, DEPT-TEGDMAwas released, and the BPO reacted with it. The addition of 0.5 wt% n-TiO_2_ increases the flexural strength, and adding 0.5 wt% chitosan increases the compressive strength of the nanocomposite, while the simultaneous use of them with 0.5 wt% has no impact on the compressive strength but improves the flexural strength by about 2%. The nanocomposite containing 0.5 wt% n-TiO_2_ and chitosan improved self-healing efficiency and K_IC-healed_ in contrast to the other sample. Water aging for 90 days versus one day had no effect on self-healing efficacy, indicating that the composite matrix was not damaged. Furthermore, the nanocomposite containing 0.5 wt% n-TiO_2_ and chitosan exhibits perfect biocompatibility. These nanocomposites have been shown to present a novel type of biomaterial.

## Funding

This research did not receive any specific funding.

## Data availability

All Data generated or analyzed during this study are included in this published article.

## Additional information

No additional information is available for this paper.

## CRediT authorship contribution statement

**Reza Ravandi:** Writing – review & editing, Writing – original draft, Investigation, Formal analysis. **Saeed Zeinali Heris:** Supervision, Methodology, Conceptualization. **Salar Hemmati:** Formal analysis. **Marziyeh Aghazadeh:** Formal analysis. **Soodabeh Davaran:** Formal analysis. **Nima Abdyazdani:** Formal analysis.

## Declaration of competing interest

The authors declare that they have no known competing financial interests or personal relationships that could have appeared to influence the work reported in this paper.
